# Diplomacia de la salud: fortalecimiento de las oficinas de relaciones internacionales de los ministerios de salud en las Américas

**DOI:** 10.26633/RPSP.2017.145

**Published:** 2017-12-05

**Authors:** Sebastián Tobar, Paulo Buss, Andrés Coitiño, Alberto Kleiman, Luiz Eduardo Fonseca, Félix Rigoli, Karen Sealey, Vanessa Victoria

**Affiliations:** 1 Centro de Relaciones Internacionales en Salud Fundación Oswaldo Cruz Río de Janeiro Brasil Centro de Relaciones Internacionales en Salud, Fundación Oswaldo Cruz, Río de Janeiro, Brasil.; 2 Organización Panamericana de la Salud Departamento de Relaciones Externas, Alianzas y Movilización de Recursos Washington, DC Estados Unidos de América Organización Panamericana de la Salud, Departamento de Relaciones Externas, Alianzas y Movilización de Recursos, Washington, DC, Estados Unidos de América.

**Keywords:** Diplomacia de la salud, cooperación internacional, cooperación técnica, recursos humanos en salud, Organización Panamericana de la Salud, Health diplomacy, international cooperation, health manpower, technical cooperation, Pan American Health Organization, Diplomacia em saúde, cooperação internacional, cooperação técnica, recursos humanos em saúde, Organização Pan-Americana da Saúde

## Abstract

*La dimensión internacional de los determinantes sociales, económicos y ambientales de la salud y sus manifestaciones impulsa a los países a emprender cada vez más negociaciones entre sí y a participar activamente en la gobernanza de la salud global y en la gobernanza global por sus inequívocas relaciones con la salud. Los ministerios de salud necesitan personal capacitado para ello. En este informe se reflexiona sobre el fortalecimiento de esa función de los ministerios de salud por medio de procesos de capacitación en diplomacia de la salud y se analiza la experiencia del Programa de Fortalecimiento de la Cooperación para el Desarrollo Sanitario (CCHD), desarrollado por el Departamento de Relaciones Externas, Alianzas y Movilización de Recursos de la Organización Panamericana de la Salud (OPS) y el Centro de Relaciones Internacionales en Salud de la Fundación Oswaldo Cruz (CRIS/FIOCRUZ). Esta reflexión parte de los participantes y de los facilitadores y coordinadores del CCHD, y se basa en la construcción de los conceptos a partir de la experiencia como soporte de la reflexión para explicar la realidad y pensar en las concepciones y prácticas de los procesos de gobernanza en salud y cooperación de los ministerios de salud. Como la diplomacia de la salud es un concepto en construcción, las experiencias de capacitación en diplomacia de la salud deben promover la reflexión crítica y dar cuenta de la identidad a partir de las concepciones y prácticas de los actores involucrados en los procesos de gobernanza global y cooperación de los ministerios de salud. En este artículo también se identifican los requisitos y los procesos de formación de recursos humanos en diplomacia de la salud*.

## Abstract (en)

Given the international dimensions of the social, economic, and environmental determinants of health and their manifestations, countries are increasingly negotiating with each other and actively participating in global health governance and global governance in general, which is unequivocally linked to health. This implies that health ministries need trained staff. This report is a reflection on how to strengthen this function in health ministries through training in health diplomacy. It analyzes the experience of the Program for Strengthening Cooperation for Health Development as part of Cooperation among Countries for Health Development (CCHD), developed by the Department of External Relations, Partnerships and Resource Mobilization of the Pan American Health Organization and the Center for International Relations in Health of the Oswaldo Cruz Foundation (CRS/FIOCRUZ). This analysis is based on feedback from participants and from facilitators and coordinators of CCHD, and it attempts to develop concepts stemming from their experiences, with the aim of explaining the current situation and reflect on the concepts and practices of health governance and cooperation between health ministries. Since health diplomacy is a concept still in construction, training experiences in health diplomacy should promote critical analysis and reflect identity, based on the conceptions and practices of stakeholders in the processes of global governance and cooperation between health ministries. This article also identifies the requirements and processes of human resources training in health diplomacy.

## RESUMO

Diante da dimensão internacional dos determinantes sociais, econômicos e ambientais da saúde e das suas manifestações, os países cada vez mais negociam entre si e participam ativamente da governança da saúde global e da governança global em geral, que está incontestavelmente relacionada com a saúde. Os ministérios da Saúde precisam ter pessoal capacitado para exercer estas atividades. Este artigo examina como fortalecer esta função dos ministérios da Saúde por meio da capacitação em diplomacia da saúde e analisa a experiência obtida com o Programa de Fortalecimento da Cooperação para o Desenvolvimento da Saúde (CCHD), desenvolvido em conjunto pelo Departamento de Relações Externas, Parcerias e Captação de Recursos da Organização Pan-Americana da Saúde (OPAS) e Centro de Relações Internacionais em Saúde da Fundação Oswaldo Cruz (CRIS/FIOCRUZ). A análise parte da perspectiva dos participantes, facilitadores e coordenadores do CCHD e busca elaborar os conceitos a partir das experiências deles visando explicar a realidade atual e pensar nos conceitos e práticas dos processos de governança em saúde e cooperação dos ministérios da Saúde. Como a diplomacia da saúde é um conceito em evolução, as experiências de capacitação nesta área contribuem para estimular uma reflexão crítica e conferir identidade a partir de conceitos e práticas dos atores envolvidos nos processos de governança global e cooperação dos ministérios da Saúde. Também são abordados os requisitos e os processos de formação de recursos humanos em diplomacia da saúde.

El contexto actual de los países de la Región de las Américas y del mundo, donde los problemas de salud trascienden las fronteras y es necesario mancomunar esfuerzos y cooperar con diversos actores para abordar y resolver muchos de los problemas de salud de la población y de los sistemas de salud, ha generado la necesidad de fortalecer las capacidades de los ministerios de salud y formar cuadros profesionales en diplomacia de la salud.

Durante las últimas décadas, hemos sido testigos de la intensificación de las interrelaciones entre la salud y otras áreas de los sectores sociales, políticos, económicos y ambientales, como el comercio, la propiedad intelectual, las finanzas, la bioseguridad o el cambio climático. El protagonismo del campo de la salud ha crecido frente al de otros campos del saber y otras instancias sectoriales e incluso se ha transformado en un espacio muy importante de producción y acumulación de capital de forma global. Ejemplo de ello es el espacio que ha ganado el complejo médico industrial y financiero en las políticas globales de salud (1).

Este fenómeno de interrelaciones se refleja a nivel nacional y en el escenario global e impone la necesidad de emprender acciones coordinadas, articuladas y negociadas (2). En la diplomacia del siglo XIX se produjeron negociaciones que asociaban enfermedades infecciosas y comercio y, con el correr de los años, la salud y las relaciones internacionales se han entrelazado cada vez más. En un mundo más interconectado, la salud adquiere un fuerte protagonismo en el escenario global, donde está emergiendo una diplomacia que negocia y consensúa compromisos internacionales sobre cuestiones relativas a la salud y sus determinantes y está cada día más presente en el intelecto de las autoridades sanitarias y en la agenda de la gobernanza global.

En este sentido, los Ministros de Salud de las Américas, reunidos en el Consejo Directivo de la Organización Panamericana de la Salud (OPS), abordaron en dos oportunidades la necesidad de desarrollar un marco conceptual común y principios rectores para fortalecer el vínculo entre la salud y las relaciones internacionales, así como la cooperación entre países para el desarrollo sanitario (3–5). Para dar respuesta a la necesidad expresada por los Ministerios de Salud de los Estados Miembros de la OPS de tener personas capacitadas que reflexionen sobre las relaciones entre las dimensiones económica, comercial y ambiental internacionales y la salud y tengan las competencias necesarias para la diplomacia y la cooperación en salud, se creó el Programa de Fortalecimiento de la Cooperación para el Desarrollo Sanitario (PFCDS), que ha tenido como principal foco a la diplomacia de la salud.

## DIPLOMACIA DE LA SALUD: DEL CONCEPTO A LA PRÁCTICA

Desde finales siglo XX hasta la actualidad, la participación de nuevos actores en la esfera diplomática ha dado origen al desarrollo de nuevos procesos y al análisis de temas sectoriales que competen a dicha esfera. De todo ello se desprende la concepción de la *diplomacia de la salud* como una serie de negociaciones desarrolladas en diversos niveles, que delinean y gestionan el ambiente de las políticas globales en salud y que, idealmente, pueden producir mejores resultados para la salud (7). Se trata de un proceso de interacción entre las partes interesadas de la salud pública y otros actores que inciden en ella y en la política con fines de representación, cooperación, resolución de conflictos, mejora de los sistemas de salud y garantía del derecho a la salud de toda la población (8).

La diplomacia de la salud desempeña un papel importante sobre todo en la gobernanza de la salud global y facilita las negociaciones que se realizan en los organismos intergubernamentales regionales y globales. Esta diplomacia de la salud se desarrolla en el ámbito de los procesos de gobernanza global entendidos como “El conjunto de instituciones, mecanismos, relaciones y procesos complejos formales o informales entre Estados, mercados, ciudadanos y organizaciones intergubernamentales o no gubernamentales, por medio de las cuales, a nivel global, intereses colectivos son articulados, leyes y obligaciones son establecidas y diferencias son mediadas” (9).

Más allá de esta definición, se pueden identificar tres categorías de interacción en torno a los temas específicos de salud pública internacional: a) diplomacia básica, negociaciones formales entre naciones, b) diplomacia multipartita, negociaciones entre naciones y otros actores, no necesariamente destinadas a alcanzar acuerdos vinculantes, y c) diplomacia informal, es decir, las interacciones entre los actores internacionales de la salud pública y sus contrapartes sobre el terreno, incluidos los funcionarios del país, las organizaciones no gubernamentales, y las empresas del sector privado y el público (10).

La diplomacia de la salud puede considerarse simultáneamente como una acción política y como un mecanismo para establecer compromisos y consensos entre los diversos actores sobre la salud y sus determinantes. Entre los actores globales que actúan sobre la salud en el escenario internacional destacan las agencias, programas y fondos de las Naciones Unidas, como la OPS/OMS, el PNUD, la FAO, el UNICEF, la OMC, la OMPI, la CEPAL, y los bancos de desarrollo y fomento. Existen, además, iniciativas de integración regional y subregional, como la UNASUR, la CELAC, el SICA o la CARICOM, que participan en los procesos de gobernanza regional y global para la salud. Otros actores son las agencias de cooperación bilateral, como la USAID o la JICA y las agencias y los mecanismos de cooperación para el desarrollo de los países de la Región, como la Agencia Brasilera de Cooperación, la Agencia Chilena de Cooperación para el Desarrollo y la Agencia Mexicana de Cooperación Internacional para el Desarrollo. Por último, también sobresale la participación de actores no estatales en las relaciones internacionales en salud, que incluyen, entre otros, a las fundaciones filantrópicas, como la de Bill y Melinda Gates, la Rockefeller o la Ford, las empresas privadas trasnacionales farmacéuticas y de alimentación que, a semejanza de otras, también influyen en la salud, las universidades, y las organizaciones internacionales de la sociedad civil, como Médicos sin Fronteras, Oxfam y Greenpeace.

 Desde un punto de vista operativo para los Ministerios de Salud, ejercitar la diplomacia de la salud implica realizar negociaciones y articular y cooperar con todos los actores teniendo en cuenta los determinantes globales y políticos para poder estructurar respuestas a los problemas de salud que trascienden las fronteras nacionales. Para ello, resulta fundamental actuar en armonía con los ministerios de relaciones exteriores y con el conjunto de actores antes mencionados que participan no sólo en la gobernanza de la salud global y regional, sino en la gobernanza global orientada a alcanzar los Objetivos de Desarrollo Sostenible. Este escenario plantea la necesidad de desplegar estrategias de capacitación y formación de personal altamente calificado para la diplomacia de la salud, lo que se ha vertebrado y plas mado en el PFCDS (6).

## EL PROGRAMA DE FORTALECIMIENTO DE LA CAPACIDAD PARA EL DESARROLLO SANITARIO

Es evidente, entonces, que el desarrollo de capacidades para la diplomacia y la cooperación en salud se ha convertido en una necesidad de los ministerios de salud de la Región. Esta necesidad se expuso explícitamente durante la Reunión Regional de Cooperación para el Desarrollo de la Salud en las Américas, que la OPS organizó en Panamá en marzo de 2015 (11).

Considerando la trayectoria de la OPS en la inclusión del enfoque de salud internacional y su papel en la gobernanza regional y global de la salud, así como la experiencia acumulada por el Centro de Relaciones Internacionales en Salud de la Fundación Oswaldo Cruz de Brasil (CRIS/FIOCRUZ) —Centro Colaborador en Salud Global y Cooperación Sur-Sur de la OPS/OMS—, se aprecia que las dos instituciones han aunado esfuerzos para desarrollar una propuesta pedagógica e implementar el PFCDS (6). Esta iniciativa se marcó como objetivo mejorar la actuación de las oficinas de relaciones internacionales en salud (las ORIS de los ministerios de salud de la Región en diplomacia de la salud y cooperación internacional) a través del análisis de la gobernanza de la salud global, la cooperación internacional y la construcción de estrategias para el fortalecimiento institucional a escala nacional. Para ello, se ha adoptado como eje conceptual la cooperación estructurante en salud, que está basada en un abordaje de “construcción de capacidades para el desarrollo” (12).

Para elaborar el PFCDS, se tuvieron en cuenta experiencias anteriores de la OPS, como el Programa de Formación en Salud Internacional (PFSI) (13) y el Programa de Líderes en Salud Internacional (14). En el cuadro 1 se resumen las principales lecciones aprendidas con estos programas y las que se consideraron cuando se elaboró el PFCDS.

El PFCDS, surgido de dos mandatos vigentes sobre salud y relaciones internacionales (3) y del fortalecimiento de la cooperación para el desarrollo sanitario (4), impulsó el desarrollo de un proceso interactivo entre los participantes basado en la experiencia y la reflexión teórica. La propuesta se elaboró tomando buena nota de las lecciones aprendidas sobre formación en salud internacional y diplomacia mencionadas anteriormente y promovió el análisis de las realidades locales como elemento de capacitación. Para ello, el método de trabajo se orientó a crear espacios de reflexión entre los agentes responsables de la ejecución de las actividades en temas prioritarios, como diplomacia en salud, la cooperación Sur-Sur, y a triangular la gobernanza de la salud global, de la salud regional y las relaciones con agencias internacionales. Además, la agenda se complementó con temas globales que son objeto de la diplomacia de la salud.

La principal consideración que se hizo al empezar a diseñar el PFCDS fue que el proceso de reflexión sobre el campo de la diplomacia de la salud global tenía que orientar las acciones hacia el fortalecimiento institucional de las ORIS o de otras estructuras de gobernanza que desarrollan esta función en los ministerios de salud. La relativa novedad de los conceptos de salud global y diplomacia y la necesidad de los países en desarrollo de adoptar una perspectiva propia impidió que se iniciara un proceso de enseñanza tradicional. Por el contrario, se empezó a desarrollar un proceso de reflexión conjunta a partir de los propios actores de los ministerios de salud y sus vivencias.

El PFCDS se ha dirigido a un público de gestores y responsables de equipos con funciones sobre la cooperación y la diplomacia de la salud de los ministerios de salud, de las ORIS o de la gestión de la diplomacia y la cooperación en salud. Por ello, se concibió como una combinación de actividades virtuales y de encuentros presenciales basados en seis módulos temáticos cuyos contenidos y competencias se describen en el cuadro 2.

**CUADRO 1.** Lecciones aprendidas del Programa Formación de Líderes en Salud Internacional

Necesidad de equilibrio entre teoría y práctica: que no sean excesivamente académicos y mantengan su pertinencia con el contexto nacional y regional en que operan. Desarrollo colectivo del aprendizaje: el poder de empoderamiento derivado de los procesos de aprendizaje colectivo es transformador y fundamental para la capacidad de liderazgo. Redes de apoyo: los participantes son apoyados en su aprendizaje por muchas personas e instituciones. Esto es esencial, dada la dedicación requerida del Programa y la complejidad del tema. Combinación entre aprendizaje virtual y presencial: articulación de las actividades virtuales y presenciales utilizando plataformas como el Campus Virtual de Formación en Salud Pública. Adaptación a los contextos en transición: es necesario ser consciente del contexto global y regional en transición y realizar cambios o ajustes de estructura y contenido.

***Fuente:***elaborado por los autores a partir de la referencia 14.

Cada uno de los primeros cinco módulos se desarrollaron con material bibliográfico y presentaciones virtuales en inglés y español. Estos módulos se prepararon específicamente para el Programa por profesionales del CRIS/FIOCRUZ, y se han puesto a su disposición a través del Campus Virtual de Salud Pública, que ha servido de plataforma de trabajo del Programa. Los facilitadores, con experiencia en las subregiones de los participantes, han acompañado el proceso desde sus inicios y han sido uno de los principales valores agregados del Programa. Su función ha sido mantener el contacto permanente con ellos para supervisar su labor en el proceso de capacitación. En el cuadro 2, se presentan las competencias que se consideran necesarias para el desarrollo de la diplomacia de la salud y la cooperación y que PFCD pretende fortalecer

## RESULTADOS PRELIMINARES DEL PFCDS

Aun cuando muchos ministerios de salud de la Región han avanzado en buena medida en la institucionalización de la función de diplomacia de la salud por medio de las ORIS, queda un importante camino por recorrer para su fortalecimiento. De la reflexión de los participantes de las ORIS en el PFCDS se plantea que las decisiones o negociaciones vinculadas con la diplomacia de la salud requieren mejorar la coordinación en los propios ministerios de salud con los ministerios de relaciones exteriores y con otras áreas de gobierno que participan en foros que influyen en la salud. En este sentido, es necesario generar mecanismos permanentes de comunicación entre estos ministerios y las misiones diplomáticas de los países en el exterior para alcanzar los niveles de coordinación óptimos.

Los ministerios de salud de los países de la Región afrontan muchos retos que deben plasmarse en una agenda internacional. Asimismo, la cooperación horizontal entre los países que afrontan desafíos comunes puede favorecer el trabajo conjunto para abordarlos desde una perspectiva solidaria y de igualdad entre ellos. Existe una gran oportunidad para construir un mapa de la oferta y la demanda de cooperación que ayude a satisfacer muchos de estos desafíos.

**CUADRO 2.** Módulos del Programa de Fortalecimiento de la Cooperación para el Desarrollo Sanitario

Módulo	Contenidos	Competencias
Módulo I. Diplomacia y cooperación en salud	Proponía avanzar en el debate y el entendimiento común de los conceptos operacionales como diplomacia de la salud, cooperación sur-sur, triangular y estructurante	1. Capacidad de analizar y conocer la influenza de los determinantes políticos globales de la salud 2. Capacidad de desarrollar e influir sobre políticas y toma de decisiones que se propician en el ámbito global, regional y nacional 3. Capacidad de conducir procesos de cambio en torno a un problema o desafío común con distintos grupos o instituciones 4. Conocimiento de los mecanismos de gobernanza de los organismos multilaterales en salud o de los que ejercen su influencia sobre la misma 5. Capacidad de desarrollar y establecer relaciones y compromisos de colaboración mutuamente beneficiosos para lograr determinados objetivos 6. Capacidad de desarrollar y comunicar información innovadora sobre la salud global 7. Capacidad comunicación y coordinación con actores sectoriales y extrasectoriales (ministerios de relaciones exteriores, misiones diplomáticas y otros ministerios)
Módulo II. Salud global: grandes desafíos contemporáneos	Propuso la reflexión de las cuestiones, problemas y desafíos contemporáneos de la salud global y regional y cómo afectan a los países	
Módulo III. Desarrollo y salud I: gobernanza de la salud global y regional	Abordó las características actuales y los problemas de la gobernanza de la salud global y regional, analizando a la OMS, la OPS y otros actores	
Módulo IV. Desarrollo y salud II: gobernanza global y regional y la salud	Analizó y reflexionó sobre las estructuras de gobernanza mundial y regional, especialmente en cuanto a su componente de salud y sus relaciones con la salud, teniendo en cuenta a las Naciones Unidas, así como a otros organismos claves y actores	
Módulo V. Salud en los procesos de integración	Analizó y reflexionó sobre la diplomacia en salud y políticas de cooperación en salud de las estructuras de integración regional y las iniciativas de cumbres en la Región de las Américas	
Módulo VI. Desarrollo de un ensayo analítico sobre la situación de la diplomacia y la cooperación en los ministerios de salud	Los participantes desarrollaron un ensayo analítico explorando cómo las ORIS o los ministerios de salud de los países actúan en el campo de la diplomacia en salud y la cooperación internacional en salud identificando los problemas que las ORIS tienen para cumplir con sus tareas y responsabilidades	

***Fuente:*** elaboración propia.

La emergencia de nuevos actores, como las organizaciones no gubernamentales y las fundaciones con mandato y agenda global, así como otras no necesariamente del ámbito sanitario, plantea la necesidad de disponer de marcos conceptuales y mapas de los intereses de los diferentes actores en los procesos de cooperación en salud que sean claros y disipen cualquier duda.

En la Región se están desplegando importantes iniciativas de integración regional con foros específicos de salud (MERCOSUR, UNASUR, CARICOM, CAN) que brindan oportunidades excelentes para identificar problemas comunes y elaborar proyectos para solucionarlos, así como para intercambiar información y lecciones aprendidas por un bloque y de apropiación por otro.

La Agenda 2030 y los Objetivos de Desarrollo del Milenio constituyen un desafío común para los ministerios de salud y en tal sentido las ORIS tienen una oportunidad para desempeñar un papel estratégico en la búsqueda de indicadores para monitorizar el trabajo intersectorial de otras instituciones que influyen en la salud, para intercambiar las lecciones aprendidas en la implementación de la Agenda entre los países, para movilizar recursos y alcanzar los ODS, y, además, para hacer un seguimiento de las desigualdades en salud en relación con estos objetivos. La reflexión colectiva de los participantes sobre los desafíos y el papel que desempeñan las ORIS se han sistematizado en los cinco ejes que se presentan en el cuadro 3.

La evaluación de la primera parte presencial se llevó a cabo encuestando de forma anónima a los participantes. Esta encuesta incluyó aspectos como la utilidad de los conocimientos, su pertinencia en relación con el trabajo de las ORIS y la de los módulos, videos y materiales desarrollados, y en ella se obtuvo una alta valoración (4,32 puntos sobre 5). Los materiales originales desarrollados para el Programa fueron luego revisados por el equipo del CRIS y tomados como insumo para redactar un libro de referencia sobre diplomacia de la salud y salud global (15).

Uno de los resultados inmediatos del Programa ha sido la elaboración de veintidós ensayos analíticos por parte de los participantes, en los cuales se han identificado no solo las políticas y los mecanismos de decisión en políticas externas y diplomacia de la salud de sus ministerios de salud, sino también los caminos que deben seguirse para su fortalecimiento institucional en diplomacia de la salud y en cooperación internacional en salud. Estos ensayos constituyen una herramienta útil para fortalecer a medio plazo el trabajo de las respectivas ORIS. Asimismo, la participación de representantes de treinta y tres Estados Miembros durante las distintas etapas del Programa ha sido un indicador de buena recepción de la iniciativa.

Sin duda, estos resultados preliminares constituyen un elemento determinante del creciente interés de los ministerios de salud en priorizar el fortalecimiento de estas funciones a través de proyectos concretos, la redefinición de sus funciones y la dotación de recursos humanos, financieros y materiales.

## CONCLUSIÓN

Lo que distingue la experiencia del PFCDS en la Región es su enfoque sobre la diplomacia y cooperación en salud, basado en un proceso de aprendizaje compartido en el cual se reflexiona críticamente sobre la propia experiencia de los dirigentes y gestores y se apunta hacia la creación de capacidades para el fortalecimiento institucional de los ministerios de salud de la Región en el campo de la diplomacia de la salud.

Dicho fortalecimiento, en lo que atañe a la diplomacia y la cooperación en salud, es una condición necesaria pero no suficiente para afrontar muchos de los desafíos de salud de la Región que trascienden las fronteras. Sin embargo, el actual contexto globalizado plantea cada día más la necesidad de disponer de una capacidad apropiada de diplomacia de la salud para poder incidir en la gobernanza global en lo que respecta a la salud y a la gobernanza de la salud global. El Programa constituye una experiencia importante para avanzar en estas conceptualizaciones.

**CUADRO 3.** Desafíos y papel de las Oficinas de Relaciones Internacionales en Salud (ORIS)

Desafíos y papel de las ORIS	✓ Necesidad de mayor alineamiento de los planes de trabajo de las ORIS con los Planes Estratégicos Nacionales tanto de salud como de desarrollo, así como con los principios de política exterior. ✓ Asociar las acciones nacionales en salud (políticas, programas e iniciativas) con los acuerdos internacionales con las ORIS como coordinadoras e intermediarias en el fortalecimiento de la política de cooperación. ✓ Potenciar la comunicación de las ORIS o los ministerios de salud con las cancillerías y misiones diplomáticas como aliados estratégicos para la toma de decisiones.
Desafíos para la cooperación entre países	✓ Crear, en articulación con los ministerios de relaciones exteriores y otros organismos gubernamentales, una estrategia de diplomacia de la salud para abordar desafíos comunes de los países. ✓ Aumentar la movilización de recursos de los países en desarrollo, reducir la dependencia de la cooperación internacional tradicional y articular de forma coordinada las ofertas de los socios internacionales.
Desafíos en la planificación de la cooperación y posicionamientos en salud	✓ Articular los planes y programas nacionales de salud con los objetivos y acciones de la cooperación internacional en salud. ✓ Mejorar la relación en intercambio de información entre el ministerio de salud y las cancillerías, otros ministerios, órganos de gobierno y agencias de las Naciones Unidas. ✓ Posicionar a la salud de forma estratégica en los marcos de gobernanza global y coordinar procesos cuyos temas tienen impacto directo a nivel global (ejemplo: Convenio Marco de Tabaco).
Desafíos en la coordinación con Naciones Unidas y el Sistema Interamericano	✓ Fortalecer las ORIS para monitorizar la coordinación con la OPS/OMS y seguir los acuerdos globales que influyen en la salud a nivel nacional, regional y global. ✓ Fortalecer la participación y la abogacía de la Región de las Américas en los cuerpos directivos de la OMS utilizando los mecanismos de coordinación existentes como, por ejemplo, el Grupo de las Américas (GRUA).
Desafíos en la articulación con los procesos de integración regional y subregional	✓ Mejorar la articulación y armonización de políticas nacionales y subregionales con el apoyo de los procesos de integración para mejorar su proyección a nivel global. ✓ Promover una relación cotidiana y directa de la OPS con los procesos de integración propiciando sinergias y complementariedad. ✓ Capacitar a las ORIS para mejorar sus habilidades de negociación con el objetivo de situar los temas de salud estratégicamente en el ámbito intersectorial.
Desafíos para el trabajo intersectorial y la Agenda 2030	✓ Desarrollar herramientas o acciones para que las ORIS apoyen a los ministerios de salud en alinear los ODS con los objetivos nacionales de salud. ✓ Apoyar a las ORIS en su función de movilización de recursos y desarrollo de alianzas estratégicas para implantar de la Agenda 2030.

***Fuente:***elaboración propia.

 En muchos países, las decisiones o negociaciones vinculadas con la diplomacia de la salud no se realizan desde una perspectiva estratégica y existe la necesidad de que las cancillerías se coordinen mejor entre ellas y con otras áreas de gobierno que participan en foros que inciden sobre la salud. La elaboración de ensayos analíticos para identificar desafíos y propuestas para fortalecer las funciones de diplomacia de la salud y la cooperación de los ministerios de salud ha supuesto una excelente oportunidad para mejorar.

Ya son varios los ministerios de salud de la Región que han dado pasos importantes en los últimos años en el desarrollo de sus funciones de diplomacia de la salud y gestión de la cooperación por medio de la creación de sus ORIS. Sin embargo, por su papel político y de alineación con las esferas gestoras de los ministerios de salud hay un grado de rotación de personal en las ORIS considerable. Así, por un lado, la coyuntura y la dimensión de los desafíos de la salud global y, por otro, los cambios frecuentes de personal en las ORIS ponen de manifiesto día tras día que es fundamental continuar fortaleciendo las iniciativas de capacitación en diplomacia de la salud.

### Agradecimiento.

Los autores expresan su agradecimiento a los participantes en el Programa, que con sus experiencias y conocimientos constituyeron el ingrediente imprescindible para el éxito de la iniciativa.

### Financiamiento.

Ninguno.

### Declaración.

Las opiniones expresadas en este manuscrito son responsabilidad del autor y no reflejan necesariamente los criterios ni la política de la *RPSP/PAJPH* y/o de la OPS.
